# A cross-sectional study of suckling calves’ passive immunity and associations with management routines to ensure colostrum intake on organic dairy farms

**DOI:** 10.1186/s13028-019-0442-8

**Published:** 2019-01-30

**Authors:** Julie Føske Johnsen, Hildegunn Viljugrein, Knut Egil Bøe, Stine Margrethe Gulliksen, Annabelle Beaver, Ann Margaret Grøndahl, Tore Sivertsen, Cecilie Marie Mejdell

**Affiliations:** 1 Department of Terrestrial Animal Health and Welfare, Norwegian Veterinary Institute, Pb 750 Sentrum, 0106 Oslo, Norway; 2 Section of Epidemiology, Norwegian Veterinary Institute, Pb 750 Sentrum, 0106 Oslo, Norway; 3 Department of Animal and Aqua Cultural Sciences, University of Life Sciences, Box 5003, 1432 Ås, Norway; 4 ANIMALIA, Norwegian Meat and Poultry Research Centre, Lørenveien 38, PB 396, 0513 Oslo, Norway; 5 Faculty of Land and Food Systems, University of British Columbia, 2357 Main Mall, Vancouver, BC V6T 1Z4 Canada; 6 Department of Production Animal Clinical Sciences, University of Life Sciences, P.O. Box 8146 Dep, 0033 Oslo, Norway

**Keywords:** Calf management, Calf welfare, Dam rearing, Failure of passive transfer

## Abstract

**Background:**

For suckling dairy calves, different management routines to ensure sufficient colostrum intake are applied: visual assessment, hand feeding supplemental colostrum or assistance. However, knowledge on the efficacy of these methods to prevent failure of passive transfer [FPT: serum immunoglobulin (IgG) < 10 g/L] is lacking. Our objectives were to explore FPT prevalence in suckling dairy calves and associations with common management routines to ensure colostrum intake. From 20 organic herds, 156 calf blood samples (mean ± SD; 7.8 ± 1.24 per herd) and 141 colostrum samples from the dams were analysed. All calves suckled the dam. Factors known to affect serum and colostrum IgG were evaluated, including the method applied by the producer to ensure calf colostrum intake and whether it deviated from routine practice for any reason.

**Results:**

The prevalence of FPT was 31%. Mean serum and colostrum IgG (± SD) were 16.0 ± 10.0 g/L and 39.4 ± 26.4 g/L, respectively. Only colostrum IgG was found to have a statistically significant influence on the prevalence of FPT. Variation in serum IgG was also explained mainly by colostrum IgG. Of calves receiving colostrum according to farm routine, calves receiving supplemental colostrum with a bottle had lower serum IgG levels than did calves receiving no additional colostrum. However, no within-herd effect was found. With a high between-herd variation, colostrum IgG ranged from 2 to 135 g/L, and only 23% of the samples had a IgG content > 50 g/L. Colostrum IgG was significantly higher in samples collected during spring, compared to samples collected during winter, and lower in 2nd parity cows.

**Conclusions:**

The results indicate that for calves capable of finding the udder and suckling independently, there is no direct benefit of routinely hand feeding colostrum although herd level factors (e.g. feeding, management etc.) may play an important role. FPT prevalence in this study was high, and comparable to that of calves in conventional herds, separating cow and calf at birth. Still, the findings of a high FPT prevalence and inferior colostrum quality indicates a need for improved awareness among dairy producers practicing cow-calf suckling.

## Background

Successful colostrum management requires that calves receive a sufficient volume of clean, high-quality colostrum within the first few hours of life. The placenta of the cow prevents the transmission of immunoglobulins (Ig) in utero [1]; consequently, calves are born agammaglobulinemic. Therefore, the calves’ immunity is fully dependent on the acquisition of adequate amounts of Ig from colostrum after birth [2, 3]. In colostrum, IgG1, one of two IgG subtypes, predominates over the other Ig’s (IgA and IgM) [4]. IgG1 (hereafter referred to as IgG) is used as a measure of colostrum quality due to its upconcentration during pre-partum colostrum formation and preferential absorption into calf serum [1]. Inadequate transfer of Ig is commonly defined by serum IgG levels of < 10 g/L at 24–48 h of age, a condition called failure of passive transfer (FPT) [5]. FPT is associated with increased mortality, as well as decreased weight gain [6–8]. Known factors influencing IgG transfer include timing of colostrum ingestion, quantity and quality of colostrum and presence of the dam [8, 9].

Ensuring sufficient colostrum intake to suckling calves is of special interest to organic dairy producers. According to national organic regulations in Nordic countries, it is mandatory to keep the calf with the dam for 1–3 days after birth [10–12]. Some studies report that calves left with the dam have higher levels of IgG absorption and serum IgG concentrations [2, 13, 14]. However, other studies have indicated that calves left with their dams after birth are at a higher risk of developing FPT [5, 15, 16]. Two intervention procedures have been described to secure colostrum ingestion in suckling calves: early assisted suckling [14, 17, 18], and manual feeding of additional colostrum to the calf [19, 20]. In a survey conducted among organic producers in Norway and Sweden, many producers stated that their routine practice is either to feed additional colostrum with a bottle, or to visually assure that the calf attains colostrum and intervene with additional measures only if the calf does not manage by itself [21].

Whereas hand feeding colostrum to calves separated from the dam is described by many authors, there is little information on the practice of feeding additional colostrum to suckling calves. The aim of the current study was to explore the prevalence of FPT in suckling dairy calves at organic farms, and associations with management strategies to ensure colostrum intake.

## Methods

### Farms and experimental design

Farms were recruited through a questionnaire distributed to the source population of certified organic dairy farms in Norway (n = 307) and Sweden (n = 210). From these, a convenience sample of 20 herds; 16 Norwegian and 4 Swedish herds were non-randomly selected based on geographic proximity to project personnel (Norway) and one selected veterinary practice (Sweden), willingness to participate and management routines to ensure colostrum (i.e. the first colostrum meal); either visual assessment (11 herds) or bottle feeding (9 herds; see below for more details). All calves in the study were left to suckle the dam during the colostrum period, defined as the first 3 days postpartum [22], and the producers were instructed to ensure colostrum corresponding to their established farm routines. Successive calvings (independent of calf sex) from each herd were included in the study population, but calves reaching sampling age of 24–48 h during the weekends were excluded because of high veterinary costs during the weekends. Otherwise, a minimum of 6 and a maximum of 10 cow-calf pairs from each herd were included, and the total number of included cow-calf pairs was 158. Of these, 10 calves were twins. Based on visual observation of the famers, only healthy cow-calf pairs were included. The study period was between October 2010 and October 2011. The mean herd size was 40 (± 16.8) cows and ranging from 15 to 65 cows. In general, the Norwegian herds were smaller than the Swedish herds (± SEM); 37 ± 1.6 (range 15–65) cows vs. 51 ± 1.6 (range 40–64) cows respectively.

The breed in all Norwegian herds was Norwegian Red cattle, while the breeds in the Swedish herds were Swedish Red and White (3 herds), or Swedish Holstein (1 herd).

### Colostrum management routines applied to secure sufficient colostrum intake to calves

The producers routinely practicing visual assessment (method hereafter called routine visual assessment) assured visually that the calf suckled or recognized that the calf had suckled by an emptied udder quarter. On farms routinely practicing to feed colostrum with a bottle, producers manually fed colostrum to the calf (hereafter referred to as routine bottle). For all routine bottle calves, the producer milked the dam and fed this supplementary colostrum to the calf with a teat bottle. Producers were instructed to ensure that all routine bottle calves received at least 0.5 L by bottle, and record the total quantity. Producers also applied non-routine methods to ensure colostrum intake. In cases where producers routinely practiced visual assessment for any reason considered that the calf was in need of additional intervention, they assisted it to reach the udder (method hereafter called non-routine assistance) or fed additional colostrum manually (non-routine bottle). The reason for applying a non-routine method rather than a routine method of ensuring colostrum was not recorded.

### Data on calves and calving

At the start of the project, both producers and the veterinarians were contacted by project personnel and were given both oral and written instructions on data collection and sampling. Throughout the study period, producers and veterinarians were urged to take contact with project personnel in case of related enquiries. For each calving, the producers and local veterinarians were instructed to record information including method of colostrum feeding and whether or not it deviated from routine practice of the herd (non-routine vs. routine), calf age at colostrum feeding (h), calf age at blood sampling (h), colostrum quantity (for bottle calves; L), colostrum quality control (yes/no), season, herd size, breed, calving difficulty (unassisted, easy pull or twins) and cow parity (Table 1). A calf girth measurement using a standard measuring tape [23] was also obtained at the time of blood sampling.

**Table 1:** Descriptive results of serum Immunglobulin G (IgG; n = 156, g/L), prevalence of failure of passive transfer (FPT, serum IgG levels < 10 g/L at 24–48 h of age) (%) and colostrum IgG (n = 141, g/L) for cow-calf pairs included in the study

	Item	Class	Serum IgG, n	Serum IgG, g/L (SEM)	FPT, % (n)	Colostrum IgG, g/L (SEM)
Factors related to method of ensuring colostrum intake evaluated in the serum IgG and FPT models	Method of ensuring colostrum intake (Routine or non-routine)	Visual assessment	61	17.9 (1.45)	31% (19)	33.9 (3.33)
		Bottle	82	15.2 (0.93)	31% (25)	45.1 (3.37)
		Assistance	13	12.9 (3.44)	54% (7)	27.9 (4.51)
	Routine method of ensuring colostrum intake?	Yes (routinely managed)	108	15.8 (1.01)	32% (35)	38.3 (2.60)
		No (non-routinely managed)	46	16.2 (1.75)	33% (15)	53.7 (6.04)
	Changed method of ensuring colostrum intake from routine?	Routine bottle	54	13.8 (1.04)	32% (17)	41.2 (3.79)
		Routine visual assessment	54	17.9 (1.56)	33% (18)	35.1 (3.5)
		Routine bottle → non-routine visual assessment	7	17.8 (4.20)	29% (2)	25.8 (8.31)
		Routine visual assessment → non-routine bottle	28	17.8 (1.75)	25% (7)	52.7 (6.04)
		Routine visual assessment → non-routine assisted	13	12.9 (3.44)	54% (7)	27.9 (4.51)
Additional predictors evaluated in the colostrum IgG model	Country	Norway	121	16.1 (0.89)	31% (38)	43.8 (2.79)
		Sweden	35	15.8 (1.85)	37% (13)	24.8 (2.37)
	Season	Winter	62	15.3 (1.35)	40% (25)	29.6 (1.93)
		Spring	52	15.7 (1.35)	31% (16)	50.7 (4.6)
		Summer	23	17.0 (2.21)	26% (6)	41.1 (4.01)
		Fall	19	18.1 (2.18)	21% (4)	36.2 (8.84)
	Breed	Norwegian Red	121	16.1 (0.89)	31% (38)	43.8 (2.79)
		SRB/SLB	35	15.8 (1.85)	30% (3)	24.8 (2.27)
	Calving difficulty	Unassisted calving	141	15.6 (0.82)	35% (49)	39.3 (2.56)
		Easy pull	5	20.4 (8.71)	40% (2)	40.5 (4.77)
		Twins	10	20.3 (2.10)	0% (0)	40.2 (3.47)
	Cow parity	1	39	15.5 (1.31)	31% (12)	43.6 (4.67)
		2	40	16.5 (1.74)	40% (16)	30.4 (3.13)
		3	36	16.2 (1.89)	31% (11)	38.3 (3.1)
		> 3	41	16.0 (1.62)	29% (12)	44.4 (5.6)
	Herd size (years cows)	15–30	62	17.7 (1.50)	27% (17)	47.2 (4.47)
		31–55	48	17.3 (1.53)	29% (14)	34.0 (3.64)
		56–65	46	12.6 (1.02)	44% (20)	35.8 (2.54)
Additional predictors evaluated in the FPT and Serum IgG models	Calf age at blood sampling (h)	24–31	52	14.2 (1.32)	37% (19)	37.6 (3.3)
		32–37	50	15.5 (1.30)	38% (19)	41.0 (4.95)
		38–55	49	18.6 (1.54)	22% (11)	37.4 (3.00)
	Missing entries	–	5	–	–	–
	Calf girth measurement (cm)	60–77	43	15.2 (1.45)	33% (14)	38.8 (4.04)
		78–81	40	17.6 (1.57)	25% (10)	47.5 (5.03)
		82–89	38	16.5 (1.85)	36% (14)	35.7 (3.9)
	Missing entries	–	35	–	–	–
	Calf age at colostrum feeding (h)	1–2	48	16.0 (1.34)	31% (15)	40.0 (3.12)
		3–4	43	15.9 (1.67)	35% (15)	42.2 (4.6)
		5–15	35	16.1 (1.65)	34% (12)	28.0 (3.04)
	Missing entries	–	30	–	–	–
	Colostrum quantity, (L, bottle only)	Low (≤ 2.0)	75	15.0 (0.98)	32% (24)	43.5 (3.39)
		High (> 2.0)	4	20.0 (2.56)	0% (0)	64.5 (15.20)
	Missing entries	–	3	–	–	–

The results are given relative to different factors evaluated as possible contributions in the statistical models. There were 141 colostrum samples analysed for IgG

*SRB* Swedish Red and White, *SLB* Swedish Holstein

### Blood and colostrum samples

Veterinarians were instructed to take blood samples from the calves. Blood was drawn from the jugular vein at 24–48 h post partum into 10 mL vacutainer tubes. The samples were sent by express mail to the Norwegian Veterinary Institute (Oslo, Norway) for analyses. Serum was extracted from the samples, and frozen at − 80 °C within 24 h after sampling.

Producers were instructed to collect colostrum from a healthy quarter as soon as possible after birth, using 20 mL plastic tubes and to freeze the samples immediately after collection. Exact timing of when the colostrum samples were taken relative to birth was not recorded. Once all colostrum samples were collected, the producers submitted the colostrum samples in cool, insulated boxes to the TINE mastitis laboratory in Molde, Norway for analyses. From farms in close vicinity to the Norwegian Veterinary Institute, the samples were collected by project personnel and thereafter submitted to the laboratory as explained above. Single radial immunodiffusion (SRID; Triple J Farms; 777 Jorgensen Place, Bellingham, WA 98226 USA) was used to determine IgG in both serum and colostrum. The diameter of the precipitation rings was measured to obtain the concentration of IgG according to test recommendations. Samples with IgG content exceeding the maximum limits of the SRID test, resulting in ring diameters outside the range of the standard reference curve, were retested after dilution according to the test recommendations. The kit’s lowest standard and detectable IgG value was 1.96 g/L. Of the collected blood samples, two were hemolysed and thus excluded.

### Statistical analyses

A total of 156 calf blood samples were analysed which corresponded to (mean ± SD) 7.8 ± 1.24 samples from each herd. Colostrum samples were analysed from 141 of the dams. Since there were 5 twin-pairs in this study, these had non-unique colostrum IgG values. The 15 missing entries in the colostrum IgG variable all occur on singletons. Consequently, 131 of non-twin calves had their consumed colostrum analysed. We had three different response variables that were evaluated by means of 3 separate regression models in order to assess levels of the calves’ passive immunity and the cows’ colostrum quality: we used FPT (yes/no; model hereafter referred to as FPT model), calf serum IgG (hereafter referred to as serum IgG model) and grams of IgG in colostrum (hereafter referred to as colostrum IgG model) as dependent variables for the three models, respectively.

Based on a priori established causal relationships, different explanatory variables were offered for each model as noted in Table 1. For descriptive purposes, each of the continuous explanatory variables were collapsed into three levels to show data from equal proportions. It was only possible to measure colostrum quantity for (routine or non-routine) bottle calves, and because of a highly right skewed distribution, this variable was dichotomised. There were missing entries for calf age at blood sampling, calf girth measurement and colostrum quantity calf (Table 1).

The method of ensuring colostrum intake was included in the model as noted in Table 1; a routine method to ensure colostrum or as a non-routine method applied by the producer when the routine method was insufficient for any reason. We also tested the specific method, whether routine or non routine (visual assessment, bottle or assisting) or an interaction of method and whether it was applied by routine or not. We used a combination of forward and backward stepwise model selection, and used the Akaike Information Criterion (AIC) for selecting the most parsimonious model. An additional (or alternative) explanatory variable was considered to significantly improve the model, if it reduced the AIC of the model by 2 or more.

In the FPT model, the associations between the explanatory variables listed in Table 1 and the likelihood that calves would develop FPT (i.e. IgG ≤ 10 g/L) was analysed using a multiple logistic regression analysis (SPSS vers. 21, IBM). The model selection evaluated the influence of the possible predictors as outlined in Table 1. A mixed effects logistic regression model including herd as a random term (using function glmer, library lme4 in R gui, vers. 3.0.3) was also considered. However, the random term failed to explain any of the remaining variation of FPT (variance of random terms estimated to zero, data not shown) and was therefore excluded from the FPT model. In the serum IgG model, we tested method of ensuring colostrum intake, colostrum IgG and other potential fixed predictors as outlined in Table 1. For this, we used a mixed effects model with herd as a random term to account for the hierarchical structure of the data (using function lme in R-library nlme). In case the method to ensure colostrum was found to have a significant effect on FPT or serum IgG, we tested if this factor explained variation only between-herds, or within-herd. For this, we performed the analyses on a subset of data: to reduce potential confounding with herds (the random intercepts) we excluded 6 herds (44 calves) that reported to have used one method only (2 routine visual assessment herds and 4 routine bottle herds). Furthermore, 4 calves were excluded because of missing data on colostrum IgG; leading to a final sample size of 14 herds and 108 calves.

For the colostrum IgG model, a linear mixed effects regression analysis was performed to assess the impact of the explanatory variables listed in Table 1. The method of ensuring colostrum intake (i.e., visual assessment and bottle) and whether it was routine or non-routine was also included to identify possible confounding of colostrum IgG although there is no direct causal path to colostrum IgG. During model selection we found that the variation caused by the random effect of herd in this model, was reduced from 25.0 to 9.5%. For models excluding the method of ensuring colostrum, AIC values increased by 3. Consequently, this variable is retained in the model and we explain possible explanations in the discussion. Herd was included as a random term. Of the 33 colostrum samples from the Swedish herds, 28 were collected during the winter. The breeds used in Norway and Sweden are different. Consequently, the effect of breed as a fixed effect (Norwegian red vs. Swedish breeds) on colostrum IgG was evaluated using a subset linear regression model containing samples from both countries taken during the winter only (this model included 28 colostrum samples from Norwegian herds).

Effects were considered significant when P < 0.05. For model validation, residuals of the selected models were plotted against the fitted values for all explanatory variables. We concluded that no major systematic patterns were present in the residuals.

## Results

The calves in this study consumed colostrum within the first 3.7 ± 2.39 (mean ± SD, n = 126) hours after birth, and most of the calves’ colostrum was ensured by routine method (routinely managed calves, n = 108; Table 1). Of these, colostrum intake was ensured either by visual assurance (n = 54) or bottle (n = 54). In 14 out of the 20 herds, and for 46 calves, the producer used a non-routine method of ensuring colostrum intake during the study period. Of calves receiving supplemental milk by bottle, either by routine or non-routine, the mean amount received by bottle (± SD) was 1.9 ± 0.55, ranging from 0.5 to 4 L and 1.26 ± 0.89 ranging from 0.5 to 4 L respectively.

### FPT

**Fig. 1:**
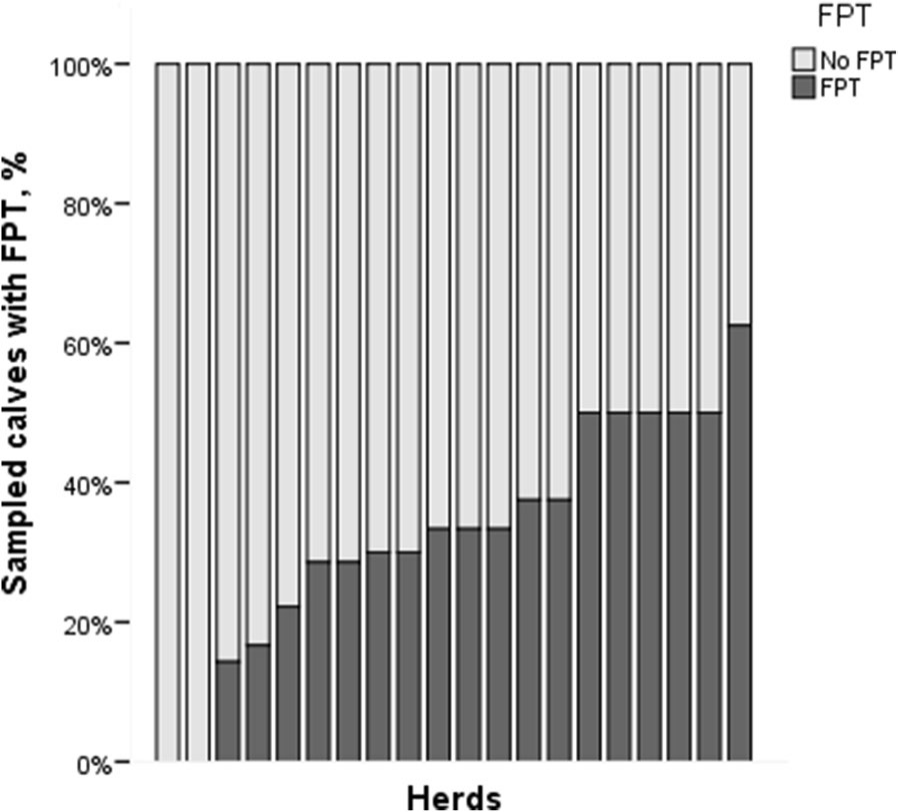
Prevalence of failure of passive transfer (FPT) among the sampled herds (n = 20). From each herd, 6–10 calves were blood sampled at the age of 24–48 h (n = 156)

Overall, prevalence of FPT was 30.8%. Between all herds, the prevalence of FPT ranged from 0 to 63% (Fig. 1). The FPT model was statistically significant, χ^2^ = (1, n = 141) = 12.10, P < 0.001 and colostrum IgG was the only significant factor to predict FPT (β =  − 0.04, SEM = 0.01, odds ratio = 0.96, P < 0.001). Method of ensuring colostrum intake was not found to predict FPT significantly (data not shown) and was not included in the model. Nevertheless, 54% of the calves for which the producer changed from routine visual assessment to the non-routine assistance (n = 13) were diagnosed with FPT.

### Serum IgG

Mean serum IgG was 16.0 g/L ± 10.03, ranging from 2 to 52.3 g/L. Colostrum IgG made significant contributions to explain serum IgG (Table 2). We also found a statistical association with method of ensuring colostrum intake. Specifically, calves for which colostrum was ensured with the routine method bottle had significantly lower serum IgG content as compared to the calves to which colostrum intake was ensured by routine method visual assessment. There was no effect of any of the non-routine methods to ensure colostrum on serum IgG levels, although the lowest values were found for non-routine assisted calves. Herd as a random effect explained < 0.0001% of the residual variance of serum IgG. AIC increased with 117 or 118 for models without method of ensuring colostrum intake and colostrum respectively.

**Table 2:** Results of the regression model where serum Immunglobulin G (g/L) was used as the response variable

Parameter	Class or mean (SD)	n	Estimate	SEM	95% CI intervals	*P* value
Intercept		141	13.82	1.38	6.474;13.801	< 0.001
Method of ensuring colostrum intake	Routine bottle	50	0			
	Non-routine bottle	26	2.86	2.38	− 1.671;7.415	0.232
	Non-routine visual assessment	7	5.28	3.94	− 2.300;12.797	0.183
	Routine visual assessment	45	4.47	2.01	0.644;8.306	0.028
	Non-routine assistance	13	0.27	3.05	− 5.609;6.061	0.939
Colostrum IgG*	39.4 (26.44)	141	0.09	0.03	0.031;0.156	0.005

*Colostrum IgG centred around the mean

A within-herd effect of method to ensure colostrum was not found when the models were rerun on a subset of data consisting only of herds where the producer had used more than one method to ensure colostrum (data not shown).

### Colostrum IgG

There was a large variation in the colostrum IgG content which ranged from 2 to 135 g/L. Only 23% of the colostrum samples had > 50 g/L IgG. Mean colostrum IgG content was 39.4 g/L ± 26.44.

The independent variables that significantly contributed to explain variation in colostrum IgG were calving season, parity and method of ensuring colostrum intake. Compared to samples from winter, colostrum IgG content was significantly higher during the spring (Table 3). Compared to 1st parity cows, colostrum IgG was lower for 2nd parity cows. Compared to dams of non-routine bottle calves, colostrum IgG values were lower for dams of routine visual assessment calves and dams of non-routine assistance calves. Herd as a random term explained 9.5% of the residual variation of colostrum IgG. Colostrum IgG (± SEM) in the cows of Norwegian Red breed (n = 28) was significantly higher than in the Swedish Red and White/Swedish Holstein (n = 28) cows (35.6 ± 2.52 vs. 23.5 ± 2.46 g/L IgG respectively); colostrum IgG subset model, n = 56, P = 0.03).

**Table 3:** Results of the regression analysis where colostrum Immunoglobulin G (g/L) was used as the response variable

Parameter	Class (n)	n	Estimate	SEM	95% CI	P-value
Method of ensuring colostrum	Routine visual assessment	45	0	–	–	–
	Non routine bottle	26	14.01	6.18	1.89;26.11	0.023
	Non routine visual assessment	7	− 2.92	10.51	− 23.52;17.68	0.781
	Non routine assistance	13	− 6.13	7.97	− 21.74;9.48	0.442
	Routine bottle	50	1.75	6.45	− 10.90;14.40	0.786
Season	Winter	56	0	–	–	–
	Spring	50	18.32	5.27	7.99;28.65	0.001
	Summer	20	9.32	7.37	− 5.13;23.77	0.206
	Fall	15	8.25	7.44	− 6.34;22.84	0.268
Parity	1	37	0	–	–	–
	2	35	− 14.04	6.06	− 25.92;2.16	0.021
	3	31	− 7.76	6.12	− 19.75;4.24	0.205
	> 3	38	− 1.54	6.04	− 13.38;10.30	0.798
Intercept		141	33.61	6.63	20.63;46.60	0.000

To evaluate whether or not samples from Norway and Sweden differed with respect to factors determining colostrum IgG and serum IgG, the models for both variables were conducted for the two countries separately, yielding similar results (data not shown).

## Discussion

We found that low colostrum IgG was the most significant predictor of serum IgG and thus FPT. Both serum IgG, and especially, colostrum IgG varied highly between herds.

Similar to other studies [5, 24, 25], this study showed that increasing colostrum IgG reduced the risk for FPT. The definition of FPT in this study (serum IgG < 10 g/L at 24–48 h) is abundantly used. However, the definition of FPT should be linked to health outcomes and thus defined in the different study populations. We encourage future research to investigate which serum IgG levels, under Norwegian conditions, are needed to protect dairy calves from disease. However, colostrum IgG in our study was well below that of other studies [26, 27]. In fact, the majority of the colostrum samples had an IgG content below 50 g/L IgG. Comparable results were found in a former Norwegian study [28]. The low colostrum quality should be taken into account when evaluating the results of the study. The prevalence of FPT was comparable to that found in a Norwegian dairy calf project where a prevalence of FPT of 30% was found in 584 randomly selected calves sampled between 1 and 7 days of age (Gulliksen, unpublished). Our findings were also comparable to that of other studies from conventional herds [29, 30]. FPT prevalence in the present study were lower than the 61% found in a study where (conventional) suckling calves were encouraged to suckle and stand [5]. On the other hand, the rates of FPT were higher than the 19% reported in an epidemiological study from conventional dairy herds [31] where most herds reported to separate cow and calf immediately post partum. The serum IgG levels in our study were also higher than what has been reported for suckling calves on organic farms [32]. Altogether, comparison of our results with other relevant studies may indicate that suckling calves receiving surveillance during the first few hours after birth have a similar risk of FPT as calves in conventional, non-organic herds that are separated from the dam and fed a fixed amount of colostrum by bottle. Nevertheless, the high FPT prevalence found in the current study implies that additional efforts need to be applied in order to increase knowledge about adequate passive transfer of immunity of suckling calves. The substantial variation in herd FPT levels, from 0 to 63%, may reflect herd variation in colostrum IgG. In fact, colostrum IgG from the cows in the two herds with no calves suffering from FPT was higher than the mean (64.7 ± 35.37 g/L vs. 39.4 ± 26.44 g/L). Initial analyses did show that mean herd colostrum IgG explained nearly as much of the variation in FPT as colostrum IgG from individual cows. However, the variation also indicates a potential to improve the management of colostrum intake of suckling dairy calves.

We could not detect an association between FPT and the method of assuring colostrum to the calf. Contributing to this lack of difference is the fact that for each calf, the different producers made the choices of which method to ensure colostrum was most suitable. Consequently, a between-herd variation in factors leading to choose a non-routine method exists. However, for serum IgG, routine bottle calves had significantly lower levels than routine visual assessment calves. Research about feeding additional colostrum by bottle to suckling calves is limited. The study of Logan [33] indicates that compared to natural suckling (without assistance), the calves´ immune status could be improved by feeding additional colostrum by hand. On the other hand, Michanek and Ventorp [34] found that calves suckling on their own within 12 h had high serum IgG levels. For the routine visual assessment calves, the producer assumed that the calf was not in need of further assistance with colostrum intake. This indicates that visually assessed calves were probably high vigour calves, which are known to consume large amounts of colostrum [34]. On the contrary, low vigour calves may need of assistance to suckle, a stratum of calves known to display an increased morbidity risk [36]. The lower serum IgG levels found in routine bottle calves do not indicate that feeding additional colostrum to calves struggling to suckle by themselves should be discouraged. The finding also reflects between-herd effects. Within herds, as analysed for herds practicing more than one method, there was no detectable effect of method of ensuring colostrum intake. Thus, the low serum IgG levels in calves receiving bottle by routine are linked to herd-level factors. Calves that received supplemental colostrum received less than the 3.5 L currently recommended in Norway [37], this likely contributed to lower serum IgG levels among the bottle calves. In these herds, routinely feeding suckling calves a (low) amount of colostrum by bottle does not seem to improve passive transfer of immunoglobulins. In addition, the extra step of harvesting colostrum from the dam may have contributed to a delay in the first colostrum intake. Although the producers may not have measured the colostrum quality from the visually assessed calves until first milking, the calves may well have obtained the colostrum in a more timely manner. Herd level factors like housing of cow and calf at calving (single maternity pen vs. group pen), calving supervision routines, infection pressure, cleanliness (colostrum, maternity pen etc.) or calf caretaker may influence on the passive immunity of calves [7, 9]. For example, Trotz-Williams et al. [38] found that in herds where the primary calf caretaker was female the calves had lower risk of FPT. Such factors were not determined in the frames of this study. Another question is whether or not a calf that has received its first meal by bottle subsequently is less motivated to seek the teat and suckle. Calf “imprinting” on the human caretaker [39] could in turn impede with how the calf associates the udder with milk. Thus, in herds routinely ensuring a minimum of colostrum intake to suckling calves by using a bottle, supervision of the continued suckling events may be of importance. In Norway, most organic producers (44%) routinely feed supplemental colostrum to their calves while 24% routinely practice visual assessment, 17% routinely assist the calves to suckle while 15% use other methods (mostly combinations of the above mentioned methods) [21]. Given that our study population was nearly equally balanced on herds routinely practicing to feed supplemental colostrum and visual assessment, the representativity of this study may be compromised by the differences between the study and the reference population.

Each farm had a specific routine practice, but we found that most farms changed method of ensuring colostrum intake to one or more of the calves during the study period. We did not record the reason for this, but expected that these calves were either in need of more (i.e. a bottle or assistance in stead of merely visual assessment) or less help (i.e. visual assessment instead of bottle). Assisting the calf to reach the udder may be considered as a “follow up” to visual assessment. In general, time available for the producer to assist each calving may also vary with herd size. In non-routinely managed calves, mean serum IgG was numerically lowest for assisted calves, and FPT rates were consequently high. Many authors have found that assisting suckling calves is effective for the absorption of IgG [14, 24, 40]. In our study, assistance was reported to be practiced instead of visual assessment upon requirement, probably because the calf did not get up and suckle by itself. The non- routine assisted calves may be the ones that fail to find the teat on their own due to e.g. large, pendulous udders with large teats or due to low calf vigour [35, 40]. It has been found that 13–45% of dairy calves were unable to suckle the dam within 6–8 h post partum [40–42]. Thus, the serum IgG levels of the non-routine assisted calves might have been even lower if the producer had not intervened. This indicates that calves identified to be in need of assistance to find the udder and suckle should receive special attention during the first months of life because of a higher risk of FPT.

Similar to the findings of Gulliksen et al. [28], colostrum IgG varied highly between herds. Herd- level factors like feeding, environment, housing and other management strategies at the individual farms are of importance with respect to colostrum quality. Organic dairy producers are mandated to restrict the usage of concentrate in favour of roughage which may affect colostrum quality. However, restricted usage of concentrate can probably not explain low IgG values in colostrum, since Gulliksen et al. [28] found a negative correlation between amount of concentrate fed to the cows and colostrum quality. The majority of the variation in colostrum IgG was explained by herd factors that were not recorded in the current study. Colostrum IgG content also varied between seasons in accordance with Gulliksen et al. [28], who found that colostrum IgG content was higher for cows calving during late summer and autumn. Similar findings were reported by Gay [43]. However, there is likely important (colostrum) management differences along the large range of herd size in our study group. As in our study, Gulliksen et al. [28] described lower IgG in colostrum from second parity cows. The results may point to a shortcoming in the management of these cows which should be addressed in future research. The colostrum samples from routine and non-routine bottle cows had a higher colostrum IgG content as compared to the dams of calves routinely visually assessed or non- routinely assisted. In this study, this explanatory variable likely represents a surrogate measure for between-herd effects that were not measured. We propose that this association between bottle feeding and colostrum IgG might be linked to the colostrum sampling. The participating producers were instructed to collect colostrum as soon as possible after birth which in practice may coincide with the time of first milking. Non-routine bottle calves were likely assessed to be in need of additional measures to attain colostrum. Bottle cows were hand milked to obtain colostrum, and the samples may thus have been collected earlier than that of routinely visually assessed or non- routinely assisted for which the producers may have awaited collection of colostrum until the first milking (information on the timing of the colostrum sample collection was unfortunately not recorded in this study). Moore et al. [44] found that colostrum samples collected 6 h after calving had a lower IgG content than colostrum collected 2 h after calving.

We found that cows of the breed Norwegian Red had a better colostrum quality than Swedish Red and White or Swedish Holstein. However, these analyses were performed on a subset of the data, with few observations. A breed difference may be attributable to genetic differences, or to dilution effects due to high milk yields as reported in other studies [9, 20]. Average yearly milk yield for Norwegian Red and that of Swedish dairy breeds is 7125 kg and 8389 kg respectively [45, 46]. Overall, the variation in colostrum quality with parity, breed and season of the year is well established [9]. The results indicate that on- farm colostrum quality control should be part of the routine colostrum management for suckling dairy calves, especially for second parity cows calving during the winter. Very low readings on an e.g. Brix refractometer indicate that supplemental colostrum should be bottle-fed.

As proposed by Flower and Weary [47] stockpersons need to ensure that suckling dairy calves attain colostrum. The results of this study indicate that no improvement in serum IgG was obtained by routinely providing supplemental colostrum to suckling dairy calves. However, colostrum quality and quantity was not standardized, and only healthy calves were included. In addition, the study sample was not taken at random, which may have led to selection bias, thus limiting the external validity of the results beyond the source population. Additionally, participation in this study was voluntary; this might have led to producers more interested in colostrum management, who are running well-managed farms, to participate. Dairy producers and veterinarians recorded data and samples. This method of collection can lead to high levels of variation, which may have contributed to the lack of significant differences between treatment groups in, e.g., the FPT model. Although both written and oral instructions were given to mitigate this variation, practical implications might have affected the sampling of colostrum, e.g. linked to calving during the night. Other interventions to improve passive transfer, e.g. bottle feeding quality-controlled colostrum at a minimum quantity of 3.5 L as soon as possible after birth may lead to improvements in FPT rates. Calves that get up and suckle on their own, generally had high serum IgG levels indicating that they are in no need of further intervention.

## Conclusions

In these study herds, the prevalence of FPT among all suckling calves was high, and comparable to that of reports from Norwegian calves in conventional, non-organic dairy herds, that are separated from the dam and fed colostrum artificially. Securing high colostrum quality is an important preventive measure of FPT in suckling dairy calves. The results indicate that for calves capable of finding the udder and suckling independently, there is no direct benefit of routinely hand feeding colostrum although herd level factors may play an important role. Herds practicing suckling need to systematically address all the three most important factors to ensure passive transfer of immunity: time from birth, colostrum quantity and colostrum quality.

## Abbreviations

FPT: failure of passive transfer
IgG: Immunoglobulin G
